# Genome Sequence of *Klebsiella pneumoniae* Urinary Tract Isolate Top52

**DOI:** 10.1128/genomeA.00668-14

**Published:** 2014-07-03

**Authors:** Jeremiah G. Johnson, Rachel R. Spurbeck, Sukhinder K. Sandhu, Jyl S. Matson

**Affiliations:** a Department of Microbiology and Immunology, University of Michigan Medical School, Ann Arbor, Michigan, USA; b Swift Biosciences, Inc., Ann Arbor, Michigan, USA; c Department of Medical Microbiology and Immunology, University of Toledo, Toledo, Ohio, USA

## Abstract

*Klebsiella pneumoniae* is a significant cause of nosocomial infections, including ventilator-associated pneumonias and catheter-associated urinary tract infections. *K. pneumoniae* strain TOP52 #1721 (Top52) was isolated from a woman presenting with acute cystitis and subsequently characterized using various murine models of infection. Here we present the genome sequence of *K. pneumoniae* Top52.

## GENOME ANNOUNCEMENT

*Klebsiella pneumoniae* is a non-motile, Gram-negative bacterium that typically exists as a commensal resident of either the human gastrointestinal tract or the nasopharynx (1). *K. pneumoniae* is also a significant cause of human disease, particularly in immunocompromised individuals and patients in long-term-care facilities (2). Primarily an opportunistic pathogen*, K. pneumoniae* can cause a variety of infections, including ventilator-associated pneumonias, catheter-associated urinary tract infections, wound infections, septicemia, and meningitis (3). Of particular concern is the observation that *K. pneumoniae* clinical isolates are increasingly resistant to multiple antibiotics. Acquisition of plasmids encoding extended-spectrum beta-lactamases has resulted in resistance to many commonly used antibiotics (4). In addition, the emergence of carbapenem-resistant strains has been observed worldwide. Carbapenem-resistant *K. pneumoniae* strains are resistant to nearly all available antimicrobial agents, and infections result in high rates of morbidity and mortality (5).

*K. pneumoniae* is the second most common cause of urinary tract infections after *Escherichia coli*, often due to the use of indwelling catheters (6). *K. pneumoniae* strain TOP52 #1721, hereafter referred to simply as Top52, was isolated from the urine of a 26-year-old woman with acute cystitis (7). After successful completion of antibiotic therapy and negative follow-up urine cultures, the patient developed recurrent cystitis with the same strain of *K. pneumoniae* as determined by restriction fragment length polymorphism (RFLP) analysis (8). Subsequent characterization of Top52 found that the organism is able to form intracellular bacterial communities and colonize the urinary tracts of mice, much like uropathogenic *E. coli*, but at lower rates (8). This is likely due to both decreased expression of the type 1 pili and variations in the fimbrial adhesion, FimH, that alter protein function (8). More recently, Top52 has also been used in a catheter-associated urinary tract infection (UTI) model of *K. pneumoniae* infection where type 1 and type 3 fimbrial mutants were found to be at a disadvantage for colonizing the urinary tracts of catheterized mice (9).

*K. pneumoniae* Top52 was grown overnight at 37°C on Luria broth (LB) agar, and genomic DNA was isolated using the Qiagen DNeasy Blood and Tissue kit (Qiagen, Valencia, CA). Genomic DNA was then fragmented to an average size of 550 bp using a Covaris M220 (Covaris, Woburn, MA). Fragments were subsequently size selected by Pippin Prep (Sage Science, Beverly, MA), and whole-genome libraries were made using the Accel-NGS 2S DNA Library kit (Swift Biosciences, Ann Arbor, MI). These libraries were then sequenced on the Illumina MiSeq (Illumina, San Diego, CA) using MiSeq Reagent kit v2. Approximately 4 million FASTQ reads were used for *de-novo* genome assembly using MIRA 4 (10), which resulted in 2,959,766 reads assembled into 85 contigs with a total consensus of 5.4 Mb (average total coverage of 83×, largest contig of 482 kb and *N*_50_ of 299 kb). A BLAST search confirmed that 45 contigs map to the chromosome and 40 are plasmid contigs.

### Nucleotide sequence accession numbers.

This whole-genome shotgun project has been deposited at DDBJ/EMBL/GenBank under the accession no. JNFE00000000. The version described in this paper is version JNFE01000000.

## References

[1] Ostfeld E Segal J Segal A Bogokovski B 1983 Bacterial colonization of the nose and external ear canal in newborn infants. Isr. J. Med. Sci. 19:1046–1049 6363353

[2] Sahly H Podschun R Ullmann U 2000 Klebsiella infections in the immunocompromised host. Adv. Exp. Med. Biol. 479:237–249 10897425 10.1007/0-306-46831-X_21

[3] Neu HC 1985 Infections due to gram-negative bacteria: an overview. Rev. Infect. Dis. 7(Suppl 4):S778–S782. 10.1093/clinids/7.Supplement_4.S778 3909337

[4] Chong Y Ito Y Kamimura T 2011 Genetic evolution and clinical impact in extended-spectrum beta-lactamase-producing *Escherichia coli* and *Klebsiella pneumoniae*. Infect. Genet. Evol. 11:1499–1504. 10.1016/j.meegid.2011.06.001 21689785

[5] Perez F Van Duin D 2013 Carbapenem-resistant *Enterobacteriaceae*: a menace to our most vulnerable patients. Cleve. Clin. J. Med. 80:225–233. 10.3949/ccjm.80a.12182 23547093 PMC3960994

[6] Ronald A 2002 The etiology of urinary tract infection: traditional and emerging pathogens. Am. J. Med. 113(Suppl 1):14s–19s. 10.1016/S0002-9343(02)01055-0 12113867

[7] Rosen DA Hooton TM Stamm WE Humphrey PA Hultgren SJ 2007 Detection of intracellular bacterial communities in human urinary tract infection. PLoS Med. 4:e329. 10.1371/journal.pmed.0040329 18092884 PMC2140087

[8] Rosen DA Pinkner JS Jones JM Walker JN Clegg S Hultgren SJ 2008 Utilization of an intracellular bacterial community pathway in Klebsiella pneumoniae urinary tract infection and the effects of FimK on type 1 pilus expression. Infect. Immun. 76:3337–3345. 10.1128/IAI.00090-08 18411285 PMC2446714

[9] Murphy CN Mortensen MS Krogfelt KA Clegg S 2013 Role of Klebsiella pneumoniae type 1 and type 3 fimbriae in colonizing silicone tubes implanted into the bladders of mice as a model of catheter-associated urinary tract infections. Infect. Immun. 81:3009–3017. 10.1128/IAI.00348-13 23753626 PMC3719564

[10] Chevreux B Wetter T Suhai S 1999 Genome sequence assembly using trace signals and additional sequence information, p 45–56 *In* Computer science and biology: proceedings of the German Conference on Bioinformatics (GCB) 99 Hannover, Germany

